# Quantification of tissue volume in the hindlimb of mice using microcomputed tomography images and analysing software

**DOI:** 10.1038/s41598-020-65214-7

**Published:** 2020-05-19

**Authors:** Alexander Wiinholt, Oke Gerke, Farima Dalaei, Amar Bučan, Christoffer Bing Madsen, Jens Ahm Sørensen

**Affiliations:** 1 Research Unit for Plastic Surgery, Odense University Hospital, Odense, Denmark; University of Southern Denmark, Odense, Denmark; 2 Research Unit for Clinical Physiology and Nuclear Medicine, Department of Clinical Research, University of Southern Denmark, Campusvej 55, 5230 Odense, Denmark; 3 Department of Plastic Surgery, Odense University Hospital, J. B. Winsløwsvej 4, Odense, Denmark; 4 Department of Nuclear Medicine, Odense University Hospital, Kløvervænget 47, Odense, Denmark

**Keywords:** Diseases, Computed tomography, Preclinical research

## Abstract

When studying illnesses that cause disturbance in volume such as lymphedema, reliable quantification of tissue volume is important. Lymphedema results in swelling and enlargement of extremities and can be both physically and psychologically stressful to the patient. Experiments in rodent models provide a cost-effective research platform and are important for preclinical research on lymphedema. When performing such research, it can be crucial to measure the changes in tissue volume. Researchers must ensure that the risk of measurement error, when measuring the tissue volume, is as low as possible. The main goal of this article was to perform a comprehensive examination of the intra- and interrater agreement and hereby assess the risk of measurement error when using microcomputed tomography (µCT) images to measure hindlimb volume. We examined the agreement between four raters with different levels of prior experience and found that the risk of measurement error is extremely low when using this method. The main limitation of this method is that it is relatively expensive and time-consuming. The main advantages of this method are that it is easily learned and that it has a high intra- and interrater agreement, even for raters with no prior measuring experience.

## Introduction

The core function of the lymphatic system is to collect excess interstitial fluid from the soft tissue and return it to the venous system^1^. Impairment of the lymphatic system leads to accumulation of fluid containing macromolecules such as proteins. The stasis of the protein rich interstitial fluid and subsequent inflammation will over time lead to chronic swelling due to adipose deposition and tissue fibrosis, this condition is known as lymphedema^2^. One of the most common problems originating from lymphedema is that the swelling of limbs or genitalia causes deformity which lowers the patients’ self-esteem and leads to poor quality of life^2,3^. Furthermore patients with lymphedema have a higher risk of infection^4^, and the complications of chronic lymphedema lead to a high cost of patient care and an increased disease burden on society^5,6^. In developed countries the most common cause of lymphedema is oncological therapy such as lymph node dissection and adjuvant radiotherapy^7^. It has been suggested that 21% of women diagnosed with breast cancer will develop lymphedema^8^. Animal models are being used to research novel treatment options for lymphedema, but the lack of standardized parameters to objectively measure lymphedema has been described as a major problem^9^. Various animal models have been used in research on lymphedema, of which the rodent hindlimb model is one of the most frequently used^10^. The rodent tail model is also frequently used but the hindlimb model has previously been described as appearing to be the most eligible and cost-effective model to investigate the reconstruction of lymphatic function^9^.

Conventional measurement techniques for hindlimb volume in mice include techniques such as water displacement^11,12^, paw thickness^13^ or circumference measurement^14,15^. These techniques are relatively cheap and easy to use, but they all have limitations. Water displacement has previously been found to yield highly varying measurements when measuring the same tail^16^. Water displacement is therefore thought to be inaccurate when measuring small volumes in mice. Any manual measurement technique such as circumferential measurement is prone to bias, especially when measuring small animals such as rodents. Paw thickness has been used as a surrogate parameter for volume of the hindlimb^13^, and has been suggested to be the most suitable method to assess the course of lymphedema in mice as it is an inexpensive, fast and reproducible method^17^. Both paw thickness and circumferential measurement are inexpensive and easy to use, but it should be noted that these methods only assess the change in volume and do not allow the researcher to directly quantify the volume of the hindlimb.

In recent studies, three-dimensional (3D) image analysing software has been used to quantify tissue volume in the hindlimb of rodents using both µCT-^17-19^ and magnetic resonance imaging (MRI)^17,20^. It has been shown that both µCT and MRI analysis provide reliable measurements of hindlimb volume when compared to conventional measuring techniques, such as caliper-measured paw thickness^17^. The limitations of using µCT and MRI scans to measure volume are that they are expensive and relatively time-consuming when compared to the alternative techniques. When such complex and expensive techniques are being considered for use, one must ensure that the techniques yield precise measurements of the hindlimb volume and that they are without great risk of measurement bias.

The aim of this study was to assess the risk of measurement bias by examining the intra-and interrater agreement^21,22^ when using µCT-scans. The population of interest consisted of C57BL/6 mice from two prior experiments, and the rater population of interest was comprised of medical staff with a BSc degree. Interrater agreement for µCT- analysis has previously been shown to be high (r = 0.975, p < 0.001)^17^. While the study by Frueh et al. focused on the comparison of µCT with several different measuring techniques, reporting additionally on interrater agreement, our study targeted intra- and interrater agreement in µCT measuring, and the Guidelines for Reporting Reliability and Agreement Studies (GRRAS)^22^ were applied. Furthermore, we investigated whether the quality of the scans would affect the intra- and interrater agreement. It was speculated that factors such as poor positioning and image quality would affect the results. Through our findings we have suggested guidelines for image quality and positioning of the mice to ensure more reliable measurements for future users of this technique.

## Results

All the mice had induced lymphedema in one hindlimb (lymphedema hindlimb) and the other hindlimb was left untouched (control hindlimb). Lymphedema was induced using a combination of radiation and microsurgery. The mice were irradiated with a dose of 10 Gray (Gy) before and after surgery. The surgical part involved ligation of three lymph vessels and extraction of two lymph nodes from the hindlimb. The mice were then µCT-scanned and the raters assessed the scans. Four raters assessed scans of 50 mice once. Two of the four raters assessed the same scans twice. Two raters had 42–56 hours of prior experience in the measuring technique (AW and FD). One rater (AB) had 16 hours of experience and the last rater (CM) was new to the technique Table 1. The scans were divided into images of high (n = 30) and low quality (n = 20) by one rater (AW). The results on the scans of high quality are included in the main article. The intra- and interrater agreement results on the scans of low quality are included in the supplementary files.

**Table 1:** Information on the raters.

Raters	AW/R1	AB/R2	CM/R3	FD/R4	AW/R1 (2^nd^)	CM/R3 (2^nd^)
Level of prior measuring experience	***	*	None	***	***	*
Time used measuring	8 hr 0 min	7 hr 15 min	13 hr 30 min	6 hr 30 min	7 hr 15 min	11 hr 15 min
Minutes used per scan	9.6 min/scan	8.7 min/scan	16.2 min/scan	7.8 min/scan	8.7 min/scan	13,5 min/scan
Duration between 1^st^ and 2^nd^ analysis	—	—	—	—	2 weeks	30 weeks

Raters are presented as R1-R4 with initials. Three asterisks indicate a high level of prior measuring experience while one asterisk indicates some experience. Time is presented as hours (hr) and minutes (min). Duration between first and second analysis is presented in weeks. The scans were reanalysed by both rater 1 and rater 3.

**Figure 1:**
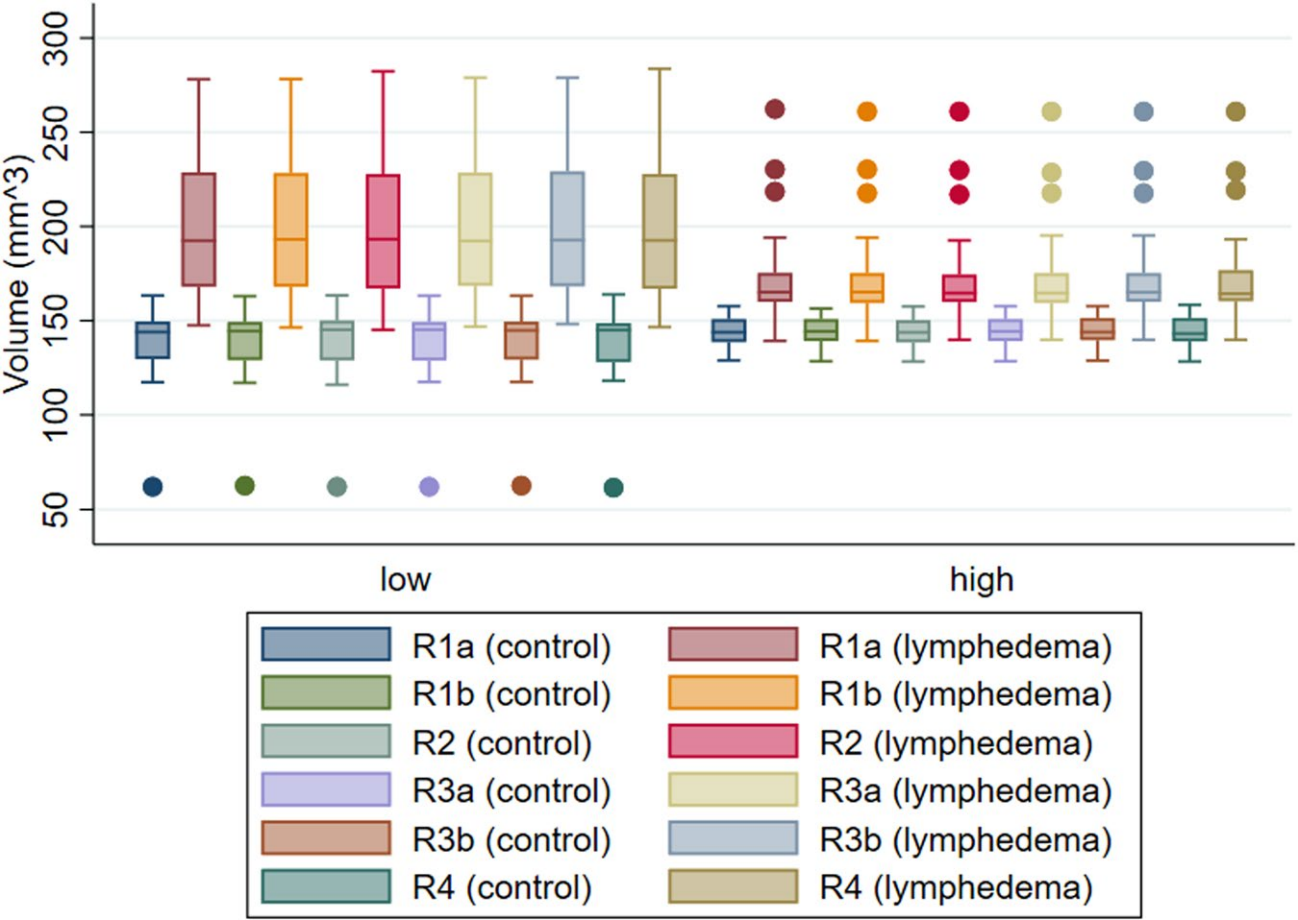
Box-and-whisker plot for hindlimb volume in mm^3^ by image quality (high (n = 30), low (n = 20)) and group (control hindlimbs, hindlimbs with induced lymphedema). Raters are presented as R1-R4. R1a indicates the first measurements of rater 1 and R1b the second measurements. R3a indicates the first measurements of rater 3 and R3b the second measurements. Boxes show first, second, and third quartiles, and whiskers represent smallest and largest observations within the fences first quartile minus 1.5 times interquartile range and third quartile plus 1.5 times interquartile range.

Measurements of scans of low quality were characterized by a wider spread than those of scans of high quality Fig. 1.

### Intrarater agreement

**Figure 2:**
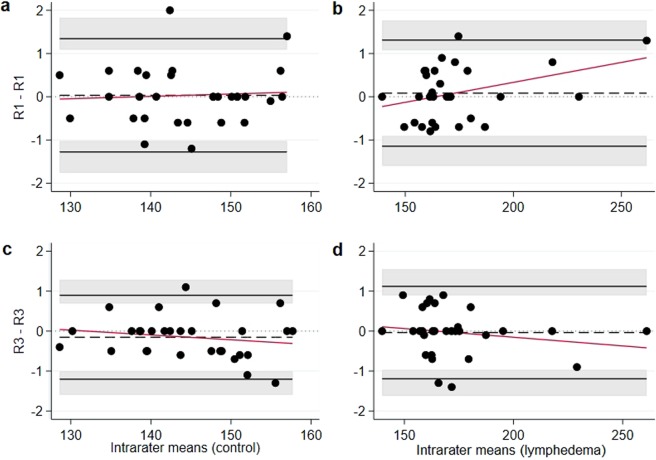
Bland-Altman plots for intrarater agreement in images of high quality (n = 30). The x-axis represents mean hindlimb volume in mm^3^, the y-axis represents intrarater differences in mm^3^. (**a**,**b**) represent data from rater 1 with “a” being data from control hindlimbs and “b” from lymphedema hindlimbs. The two analyses were performed with an interval of 2 weeks. (**c**,**d**) represent data from rater 3 with “c” being data from control hindlimbs and “d” from hindlimbs with induced lymphedema. The two analyses were performed with an interval of 30 weeks. The grey areas represent 95% confidence intervals for the Limits of Agreement. The red line is a linear regression line of the differences on the means.

The mean difference for repeated measurements of the control hindlimb was larger for scans of low quality (0.29 mm^3^) than for those of high quality (0.03 mm^3^), whereas these differences were 0.08 mm^3^ for lymphedema hindlimb independently of scan quality for rater 1 Table 2. For rater 3, the mean difference for repeated measurements of the control hindlimb was, on the contrary, independent of scan quality (−0.14 mm^3^, −0.15 mm^3^), whereas these differences were −0.37 mm^3^ and −0.04 mm^3^ for scans of low and high quality, respectively, for lymphedema hindlimb. All estimated differences were, therefore, close to 0. The variability of repeated measurements was similar for control and lymphedema hindlimb, leading to Bland-Altman Limits of Agreement (BA LoA) roughly ranging between −1.3 and 1.5 mm^3^ (with outer confidence limits between −1.76 and 1.83 mm^3^) for scans of high quality Fig. 2 (for scans of low quality, BA LoA ranged from −2.23 to 1.50 mm^3^, with outer confidence limits between −3.17 and 2.44 mm^3^ ***(***Supplementary Fig. S1***))***. The BA plots suggested symmetrical spread of paired differences for scans of both low and high quality; two measurements of rater 1 in the upper measurement range exceeded the values of the second rating in high quality scans. Moreover, the variance of paired differences appeared to be homoscedastic over the whole measurement range for both low and high quality scans.

**Table 2:** Estimated mean difference, standard deviation and Bland-Altman Limits of Agreement for intra- and interrater comparisons.

Comparison	All images (n = 50)		Images of low quality (n = 20)		Images of high quality (n = 30)	
	Control	Lymphedema	Control	Lymphedema	Control	Lymphedema
R1-R1	0.13 (0.66)	0.08 (0.64)	0.29 (0.62)	0.08 (0.69)	0.03 (0.67)	0.08 (0.63)
	−1.15, 1.42	−1.18, 1.34	−0.93, 1.50	−1.27, 1.42	−1.28, 1.34	−1.15, 1.31
R3-R3	−0.150.15 (0.57)	−0.17 (0.76)	−0.14 (0.62)	−0.37 (0.95)	−0.15 (0.54)	−0.04 (0.59)
	−1.26, 0.96	−1.67, 1.33	−1.36, 1.09	−2.23, 1.50	−1.20, 0.90	−1.19, 1.12
R1-R2	0.03 (0.88)	0.12 (1.13)	0.14 (1.09)	0.05 (1.56)	−0.03 (0.72)	0.16 (0.76)
	−1.68, 1.75	−2.10, 2.34	−1.99, 2.26	−3.00, 3.10	−1.44, 1.37	−1.32, 1.65
R1-R3	−0.06 (0.60)	−0.02 (0.84)	0.02 (0.66)	0.03 (0.95)	0.12 (0.55)	−0.04 (0.78)
	−1.23, 1.10	−1.67, 1.64	−1.29, 1.32	−1.83, 1.88	−1.20, 0.96	−1.58, 1.49
R1-R4	0.10 (0.95)	0.16 (1.56)	0.26 (0.99)	−0.03 (1.98)	0 (0.92)	0.28 (1.23)
	−1.75, 1.96	−2.91, 3.22	−1.68, 2.20	−3.91, 3.86	−1.80, 1.80	−2.13, 2.69
R2-R3	−0.10 (0.82)	−0.13 (1.10)	−0.12 (0.94)	−0.03 (1.34)	−0.08 (0.74)	−0.21 (0.93)
	−1.70, 1.50	−2.29, 2.02	−1.97, 1.73	−2.65, 2.60	−1.52, 1.36	−2.02, 1.61
R2-R4	0.07 (0.97)	0.04 (1.29)	0.13 (1.09)	−0.08 (1.12)	0.03 (0.91)	0.12 (1.41)
	−1.84, 1.98	−2.49, 2.57	−2.00, 2.25	−2.26, 2.11	−1.75, 1.81	−2.64, 2.88
R3-R4	0.17 (0.86)	0.17 (1.57)	0.25 (0.64)	−0.05 (1.70)	0.12 (0.98)	0.32 (1.49)
	−1.51, 1.85	−2.91, 3.25	−1.01, 1.50	−3.39, 3.29	−1.81, 2.04	−2.59, 3.24

Raters are presented as R1-R4, each line comparing the measurements of one rater vs another. Data is divided into “All images”, “Images of low quality” and “Images of high quality” and further subdivided into data from the control hindlimbs (Control) and the hindlimbs with induced lymphedema (Lymphedema). Data is presented as: estimated mean difference and (standard deviation) both in mm^3^ with the Bland-Altman Limits of Agreement below also in mm^3^. The Bland-Altman Limits of Agreement is the mean difference ± 1.96 SD of the difference.

### Interrater agreement

**Figure 3:**
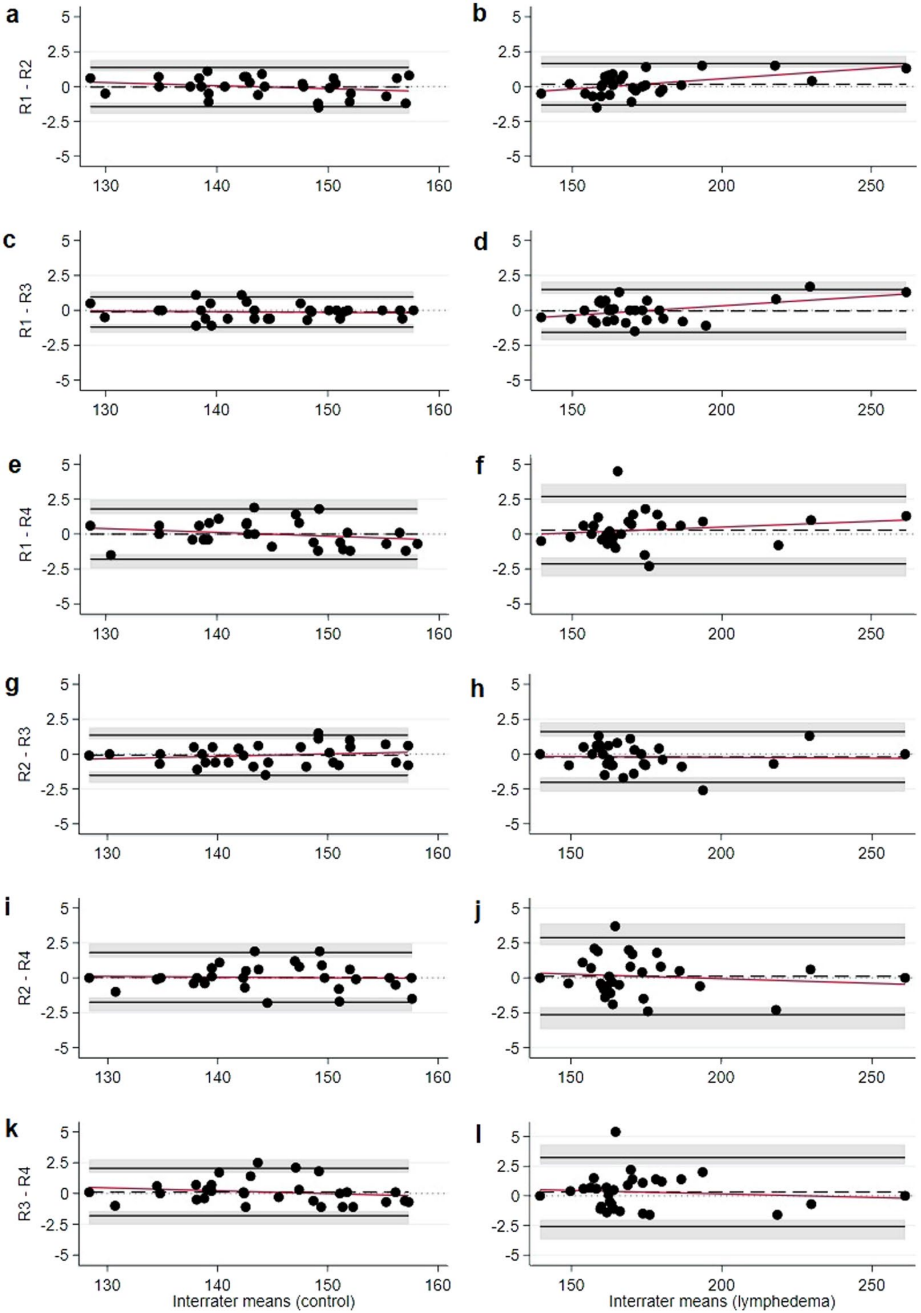
Bland-Altman plots for interrater agreement in images of high quality (n = 30). The x-axis represents mean hindlimb volume in mm^3^, the y-axis represents the interrater difference in mm^3^. R1-R4 represent the four raters. Figure 4a,c,e,g,i,k show data from control hindlimbs. Figure 4b,d,f,h,j,l show data from hindlimbs with induced lymphedema. The grey areas represent 95% confidence intervals for the Limits of Agreement. The red line is a linear regression line of the differences on the means.

The maximum estimated mean difference between pairs of raters was 0.26 mm^3^ for control hindlimb and 0.08 mm^3^ for lymphedema hindlimb in scans of low quality Table 2. In scans of high quality, it was 0.12 mm^3^ for control hindlimb and 0.32 mm^3^ for lymphedema hindlimb. The variability of paired differences was larger for lymphedema (low quality: 0.95–1.98; high quality: 0.76–1.49) than control hindlimb (low quality: 0.64–1.09; high quality: 0.55–0.98) and most of the time larger for scans of low quality than for scans of high quality (exceptions were R3-R4 for control hindlimb and R2-R4 for lymphedema hindlimb Table 2. The widest BA LoA across all interrater comparisons for control hindlimb were −2 to 2.25, with outer confidence limits −3.07 and 3.32 (low quality) Supplementary Fig. S2 and −1.81 to 2.04, with outer confidence limits −2.52 and 2.75 (high quality) Fig. 3k; for lymphedema hindlimb, these were −3.91 to 3.86, with outer confidence limits −5.87 and 5.82 (low quality) Supplementary Fig. S2 and −2.59 to 3.24, with outer confidence limits −3.67 and 4.31 (high quality) Fig. 3l. Three measurements of rater 1 in the upper measurement range exceeded the values of raters 2 and 3 Fig. 3b,d. Consequentially, BA LoA were wider for lymphedema than control hindlimb for scans of both low quality Supplementary Fig. S2 and high quality Fig. 3, and low quality scans implicated wider BA LoA than high quality scans. Again, the BA plots suggested symmetrical spread of paired differences for scans of both low and high quality, and the variance of paired differences appeared to be homoscedastic over the whole range of measurements for both low and high quality scans.

## Discussion

In this study we examined the intra- and interrater agreement when measuring hindlimb volume using µCT-scans and analysing software. We examined the interrater agreement between four raters with different levels of prior measuring experience. We found that the technique has an extremely low risk of measurement bias, even for users with no prior experience. The mean hindlimb volume of all hindlimbs previously measured was approximately 150 mm^3^. When planning the project, it was decided that if the raters’ measurements differed with less than 5% of the mean hindlimb volume, we would consider the technique to have a low risk of measurement bias. Our pre-set expectation was therefore that the maximum estimated mean difference between pairs of raters would be less than 7.5 mm^3^. The results show that the maximum estimated mean difference between pairs of raters (0.26 mm^3^) was much lower than we expected. The measuring technique can be performed by anyone who has received basic training and the technique is easily taught. We found that a person with a basic understanding of CT imaging e.g. a Bachelor of Science in Medicine, could be taught the technique in approximately one hour and only needed minor supervision during the first four trial measurements. The results show that after the technique has been taught, the level of interrater agreement is high no matter the level of measuring experience. We also found that the intrarater agreement is high even after 30 weeks of not using the technique ***(***Fig. 2c,d***)****.* This indicates that once the technique has been taught it can easily be picked up after several months, with no need to relearn the technique and minimal risk of measuring bias. Furthermore, we found that scans of low quality do influence the interrater agreement. The low quality scans had a wider spread of measurements than the scans of high quality ***(***Fig. 1***)***. The scans of low quality also had the largest mean difference in intrarater agreement and had wider BA LoA than high quality scans in interrater agreement. Although it is interesting to note that the scans of high quality all had a greater estimated mean difference when measuring the lymphedema hindlimb than the scans of low quality ***(***Table 2***)***.

A weakness of this study is that all raters were aware that their measurements would be compared with measurements of other raters. The raters’ behaviour when measuring could have been altered by the knowledge of being observed i.e. a Hawthorne effect^23^. Another weakness is that all raters were taught the technique by the same person (AW). To our knowledge the measuring technique used in this article does not have a step by step tutorial for new users and therefore it is best taught by someone who already knows the technique. Furthermore, it is also noteworthy that there are various software packages available for measuring volume through µCT-imaging and that this article only examines the intra- and interrater agreement when using Inveon Research workplace software. We cannot guarantee that using different software will yield as high intra- and interrater agreement. Another weakness is that the population of raters is relatively small. In future research a more comprehensive study with a higher number of raters could be performed. Even though more raters could have been included, we still present the interrater agreement of several raters with different levels of experience, which to our knowledge has not previously been done. A strength of this study is that the Guidelines for Reporting Reliability and Agreement Studies have been applied. The guidelines were derived by consensus of experts on intra- and interrater agreement and reliability studies to ensure the best possible reporting of agreement and reliability studies.

By applying the guidelines our study differs from previous studies in several aspects. To our knowledge, no previous study has described the agreement between several raters with different levels of prior experience in measuring. Additionally, we have included how long it takes to learn the measuring technique, under what conditions and how the measurements were performed. We have also included how good positioning affects interrater agreement compared to poor positioning, and we examined the intrarater agreement. Frueh et al. had shown that the interrater agreement was high (r = 0.975, p < 0.001) for µCT analysis^17^. Their BA LoA where a lot wider than ours when examining the interrater agreement. This was probably due to using the gluteal skinfold as a landmark when measuring the interrater agreement. They subsequently discovered that using the tibio-fibular joint as a landmark yielded higher correlation between the different 3D modalities. Our results support Frueh *et al*.’s statement that it will be reasonable to limit 3D limb volumetric to distal parts of the extremities^17^.

**Figure 4:**
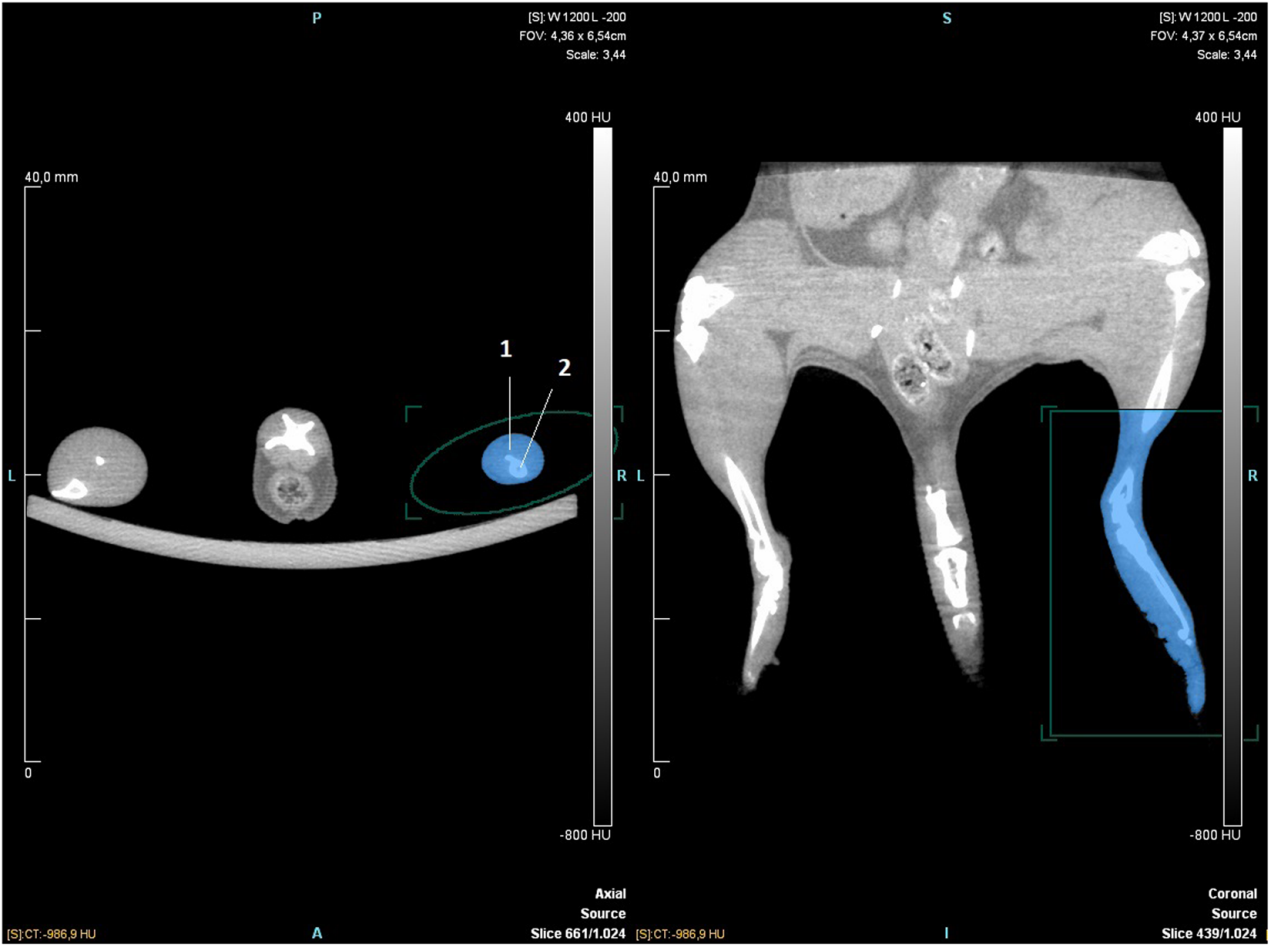
Example of mouse in good positioning. The mouse is shown in axial and coronal view. (1) indicates Fibula, (2) indicates Tibia. The mouse is seen in prone position. The axial view shows the tibio-fibular joint and the start of the ROI. The green circle is the ROI template and the blue colour indicates the tissue that is included in the ROI.

**Figure 5:**
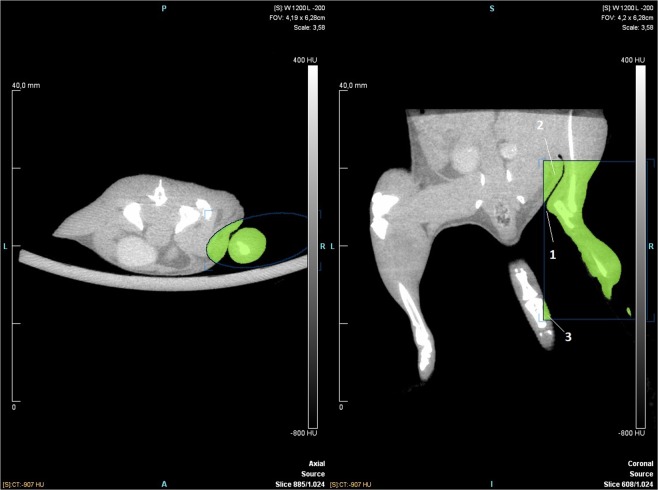
Example of mouse in poor positioning. The mouse is shown in axial and coronal view. (1) Indicates the medial point of the hindlimb, (2) indicates irrelevant trunk tissue, (3) indicates irrelevant tail tissue. The mouse is seen in prone position. The axial view shows the tibio-fibular joint and the start of the ROI. The blue circle is the ROI template and the yellow colour indicates the tissue that is included in the ROI.

The results of this study show that this technique has a low risk of measuring bias. The maximum estimated mean difference between pairs of raters was 0.26 mm^3^ which is 0.17% of the mean hindlimb volume (150mm^3^). This study might help researchers decide whether to use µCT for measuring hindlimb volume in mice. While being expensive and time-consuming, especially for raters new to the technique ***(***Table 1***)***, the method has a very low risk of measuring bias and measures the volume of the hindlimb directly. Furthermore, we have shown that the positioning is important, when measuring the hindlimb volume. The variability of paired differences was predominantly larger for scans of low quality than scans of high quality, and consequently low quality scans implicated wider BA LoA than high quality scans. We found that the quality of the scan mainly depended on the positioning of the mouse. In some cases, the borders of the bones were a little blurry due to erratic movements of the mouse, during the scan. It was speculated that the blurriness could make it difficult for the rater to find the exact point where tibia and fibula joins. However, the contrast and lighting of the scans can be altered in the analysing program so that the tibio-fibular joint clearly can be visualized in cases of doubt. We therefore found that when using the settings for the µCT-scanner described in the “Methods” section, the main factor determining the quality of the scans was positioning of the mouse. Figure 4 is an example of a high quality µCT-scan. The hindlimb is stretched so that the Region of Interest (ROI) template can easily be placed around the hindlimb. The ROI does not include irrelevant tissue and therefore only hindlimb volume gets measured. Figure 5 is an example of a low quality µCT-scan. The hindlimb is not stretched and the ROI template cannot be placed so that it covers the medial point of the hindlimb without also covering irrelevant tissue. The ROI therefore includes part of the trunk. The tail is not lying straight down and is therefore also included in the ROI. In cases like these, the rater has to manually erase the irrelevant tissue from the ROI which increases the risk of measurement bias. We therefore recommend that both hindlimbs are stretched as much as possible and that the tail is lying straight down when performing the µCT-scan as seen in Fig. 4. This will ensure the best possible conditions for measuring hindlimb volume.

In future studies it might be possible to blind the raters so that they are not aware of being observed. In future research it would also be interesting to apply the measuring technique to the rodent tail model as this model is also well established and frequently used for inducing lymphedema in mice and rats. It would also be interesting to compare the measurements obtained by human raters with automated image analysis or machine learning. The µCT measuring technique lacks a step by step tutorial so that it can be easily taught. It would be interesting to investigate whether a rater who was only taught the technique by a step by step tutorial would have the same high interrater agreement. Our department plans on making a visualized article describing the measuring technique in detail, so that the technique can be replicated as precisely as possible by other research centers.

## Methods

### Animals

C57BL/6 mice from Janvier (Janvier Labs, Le Genest-Saint-Isle, Saint-Berthevin Cedex, France) were used. The mice were inbred for more than 20 generations. The mice were acclimatized for seven days after arrival to the University of Southern Denmark Animal Care Facility. Postoperatively the mice were housed individually and received oral analgesic treatment (Buprenorphine, 0.2 mg/g) daily for three days. On the fourth day they were transferred to cages of 3–8 mice. They were maintained at a normal 12-hour day/night cycle at 21 degrees Celsius with a humidity of 45–55%. They were fed a standard diet and had access to water ad libitum. The mice were euthanized by cervical dislocation under anaesthesia at the end of the study.

All mice were female with a bodyweight of 16–18 g. The mice from both prior experiments were housed in the University of Southern Denmark Animal Care Facility as per institutional guidelines. All procedures involving animal subjects have been approved by The Animal Experiments Inspectorate, Ministry of Environment and Food of Denmark. The experiments were conducted in accordance with the European legislation on the protection of animals (Directive 2010/63/EU).

### Lymphedema model

All mice had induced lymphedema in one hindlimb and the other hindlimb served as control hindlimb. The sample of mice is divided into two groups “High quality” and “Low quality” each with two subgroups “lymphedema hindlimb” and “control hindlimb”. The model that was used for inducing secondary lymphedema has previously been described^18^. The model comprised three steps:

(1) Pre surgery irradiation (7 days before surgery): The mice were anesthetized using a vaporizer set up with 3% isoflurane (ScanVet Animal Health, Fredensborg, Denmark) with an oxygen flow rate of 0.8 − 1.2 L/min. When a mouse was fully anesthetized it was placed in supine position under the source of radiation (D3100 Gulmay, Surrey, United Kingdom). A 1.5 mm thick lead pad was then placed on the hindlimb. The lead pad had a circular hole with a diameter of 25 mm. This was placed to ensure that only the part of the hindlimb that later would undergo surgery would be irradiated. The mouse would then get a dose of 10 Gy radiation at a dose rate of 5.11 Gy/min (100 kVp, 10 mA). It is important to note that safety precautions must be taken when working with radiation. During this experiment, radiation was performed in a room built to minimize x-ray contamination. The source of radiation was only turned on when all personnel had left and sealed the radiation insulated room.

(2) Surgery: Before surgery the mice were anesthetized using a mixture of 1 mL of fentanyl (0.315 mg/mL, Hameln pharma plus, Hameln, Germany), 1 mL of midazolam (5 mg/mL, Hameln pharma plus, Hameln, Germany), and 2 mL of sterile water (Mediq Danmark, Brøndby, Denmark). The dose was 0.1 mL of anesthetic per 10 g of mouse bodyweight and was injected subcutaneously as a bolus injection. When the mouse was fully sedated, the hindlimb chosen for the procedure was shaved using electrical clippers. A circular incision was then made approximately 5 mm proximal to the popliteal fossa. Then the skin was gently blunt dissected so the area distal to the knee was clearly visible. The mouse was placed in prone position and approximately 0.01 mL of patent blue V (25 mg/mL, Guerbet, Villepinte, France) was injected between the second and third toe using a Microfine U-40 syringe (0,5 ml, Becton Dickinson, Wokingham, Great Britain). The lymph vessels and lymph node were then colored by the patent blue V and could be clearly visualized. The important structures were a lymph node located in the popliteal fossa, a proximal lymph vessel and two distal lymph vessels. All the lymph vessels could be found adjacent to the ischiatic vein. The lymph vessels were magnified using a microscope with a magnification range of 4 × −25 × (Opmi pico microscope F170, Zeiss, Oberkochen, Germany). When the vessels were clearly magnified, they were ligated using 10–0 nylon suture (S&T, Neuhausen, Switzerland). The proximal lymph vessel was ligated first, then the two distal lymph vessels were ligated. After that, the popliteal lymph node was removed using microscope and microsurgical tools (Lawton, Fridingen, Germany). The inguinal fat pad was then removed while cauterizing any vessels running through it. Resecting the inguinal fat pad is the easiest way to remove the inguinal lymph node, as the lymph node can be difficult to differentiate from the fat. Then the hindlimb was rinsed thoroughly with sterile saline and it was confirmed through the microscope that any small hairs and particles had been removed from the surgical area to avoid wound contamination and infection. The skin edges were sutured down to the muscle facia using 6–0 nylon suture (B Braun, Frederiksberg, Denmark), leaving a gap of 2–3 mm to constrain the superficial lymph flow. Previous research has shown that a temporary skin gap is often needed to mimic the human wound healing process^24^. Postoperative analgesia consisted of 0.2 mL of buprenorphine (0.3 mg/mL, Temgesic, Indivior, Berkshire, Great Britain) mixed with 2 mL of saline. The dose administered was a bolus subcutaneous injection 0.02 mL analgesia which was given 3 times daily for 3 days. The mice were given individual cages to recover.

(3) Post surgery irradiation (3 days after surgery) The procedure is described under pre surgery irradiation.

For the exact guide on the lymphedema model we refer to a separate article^25^.

### µCT scans

The scans chosen for analysis stem from a pool of approximately 576 scans of 81 mice. The mice were part of two prior experiments in which secondary lymphedema was induced in one hindlimb of each mouse using the previously described method^18,25^. During these experiments µCT scans were made every week after surgery to measure the hindlimb volume of the lymphedema hindlimb and control hindlimb. The first µCT scans of the mice were conducted one week after surgery. The mice were scanned again at two and three weeks after surgery continuing up to eight weeks after surgery. The 45 mice from the first experiment were µCT scanned for eight weeks, whereas the remaining 36 mice from the second experiment were scanned for six weeks. The scans used in this article were chosen from the 576 scans by random selection generated by a computer. A random number was generated for every scan and the scans were then placed in numerical order according to their given number. The first 50 scans where then chosen for analysis. Of the initial 50 scans chosen by random selection, three scans could not be analysed. The three subsequent scans from the randomly generated list were then chosen to replace them. AW chose whether these scans belonged in the “High quality” or “Low quality” group. Scans qualified for the “Low quality” group when the borders of the bones were blurry due to erratic movements or when the hindlimb was lying to close to other kinds of tissue. For example: when the hindlimb lies to close to the trunk, it results in inclusion of irrelevant tissue as Fig. 5 depicts. The raters then have to manually remove the irrelevant tissue. Scans qualified for “high quality” on two parameters. The first parameter was that the border of the bones had to be clear and distinct so that the tibiofibular joint could clearly be visualised when measuring. The second parameter was that the hindlimbs had to be well positioned so that the rater could easily measure the hindlimb volume, without including irrelevant tissue as Fig. 4 depicts. As described in the discussion section we found that the most important factor for good quality scans is good positioning of the mice.

**Figure 6:**
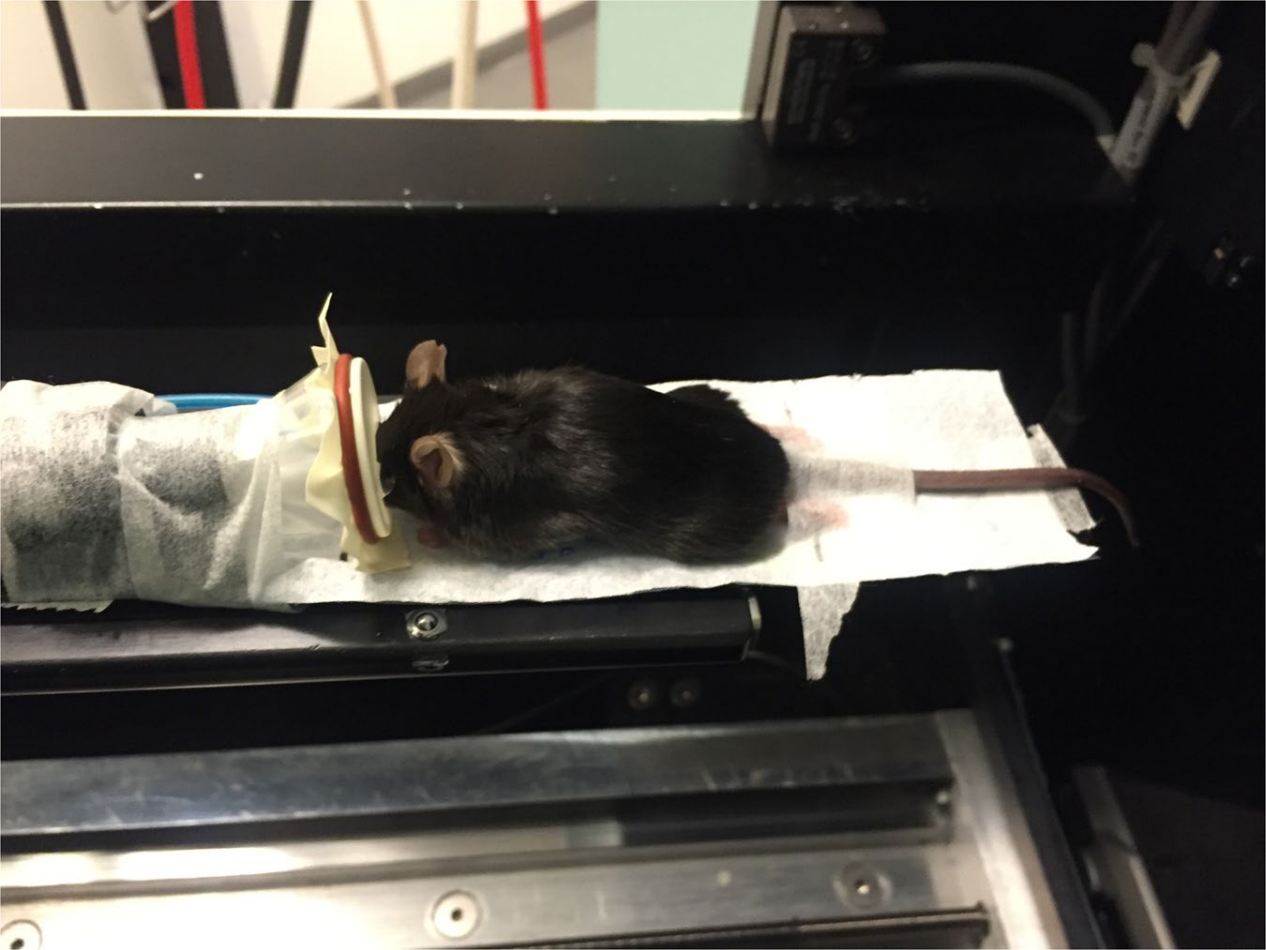
Example of a mouse positioned in the µCT-scanner. The mouse is shown lying in prone position. The hindlimbs and tail are stretched and fixated with surgical tape.

The µCT-scans were performed on a Siemens INVEON multimodality pre-clinical scanner (Siemens pre-clinical solutions, Knoxville, TN, USA). During each imaging session, the animals were anesthetized with 1.5–2% isoflurane (ScanVet Animal Health, Fredensborg, Denmark) mixed with 100% oxygen. The mice were placed front feet first in prone position on a heated animal bed (38 mm) as depicted in Fig. 6 *and* Supplementary fig. S3-S4. CT-scans were performed with a standard camera in rat mode and 1.0 mm aluminium filter with the following settings; 360° rotation with 360 projections and 2×2 bin. The magnification was set at low-medium, yielding an isotropic pixel size of 47.83 µm and a transaxial field view of 49 mm in a single bed position. Tube voltage was set to 80 kV, current was 500 µA and each projection was exposed for 1200 ms. CT scans were reconstructed using Hounsfield calibrated Feldkamp algorithm, with Sheep-Logan filter and slight noise reduction, no down sampling and 4 iterations. A scan of one mouse took approximately 11–12 minutes including time for anaesthesia. The scans included tail and hindlimbs and ended proximal to the hindlimbs Figs. 4 and 5.

### Measuring

The analysis of the scans was performed in INVEON research workplace software, version 4.2 (IRW; Siemens Healthcare, Ballerup, Denmark), using the same computer for every analysis. The software allows the user to measure tissue volume in a manually selected area. All the measurements were performed on the same computer in a standard office under no special conditions e.g. ambient lighting. It has been shown that a standardized anatomical landmark is crucial to obtain reliable measurements, and therefore the tibio-fibular joint was used as previously described^17^. The µCT-images were transferred to the computer containing the INVEON research workplace software using an USB-stick. When the software was started the raters clicked “Administration” in the top left toolbar and selected “Database” in the dropdown menu that appeared. A new dialogue box appeared. By clicking “Browse” the raters could select the folder containing the images that was going to be analyzed. The images were analysed using the “General analysis” function. The program allowed the raters to view the µCT-scans in “axial”, “coronal” and “sagittal” view simultaneously. To adjust the positioning of the images the raters used the “Pan the view” function. It is important to note that when only images are provided for commands in the program, hovering the curser above the image, will provide the name of the command. The rater moved through the images using the “Show crosshairs on all views” function. The raters located the tibio-fibular joint in the axial view of the µCT scans. They then defined a Region of Interest (ROI) template using the “Create an ROI from a template” function under the “Create” tap. The ROI started at the tibio-fibular joint and included all tissue distal to the joint. The ROI template was chosen to be cylinder shaped. The raters enhanced the cylinder-shaped ROI so that it covered the hindlimb. The raters then made sure, that the ROI template included all hindlimb tissue below the tibio-fibular joint and that it did not include any irrelevant tissue. The ROI could be made bigger or smaller in different dimensions by pulling the gray squares surrounding it. The grey circle connected to the circle in the middle of the ROI, could be used to rotate the ROI. To make sure that the ROI ended exactly at the distal tibio fibular-joint the curser was placed in the axial view and the mouse scroll wheel was used to scroll one image at a time. This allowed the researcher to find the exact image where tibia and fibula were tangent to each other. When that exact spot had been found the rater looked at the coronal view. The horizontal line of the crosshairs would then represent the exact spot of the distal TF-joint. The raters then adjusted the upper border of the ROI so that it lined up with the horizontal line of the crosshairs. The Hounsfield range was set from −500 to 4000 Hounsfield Units (HU) using the “Perform ROI thresholding” function. This option in the software allows the users to set a Hounsfield threshold for the area of tissue they want to measure. The software then quantified the tissue volume within the ROI and all voxels within the Hounsfield-interval of −500 HU and 4000 HU were included in the hind limb volume. The new ROI with a Hounsfield threshold could now be viewed, and the measured volume of the hindlimb was presented in the field below the images. If the µCT-scan was of low quality and the mouse was positioned poorly, the ROI template would sometimes include irrelevant tissue as seen in Fig. 5. If the ROI template included irrelevant tissue the raters had to erase the irrelevant tissue from the ROI manually using the “Use the eraser tool” function found under the “Edit” tap. Different shapes and sizes of erasers can be found, it is important to note that the “Cuboid” and “Sphere” shapes removes areas in 3 dimensions. We found that it is easier to include too much tissue in the ROI and then erase some (rather than including too little and add some). Inveon Research Workplace has a “Paintbrush” function that lets the user add tissue to the ROI but this function does not take the Hounsfield threshold into account and will usually result in inclusion of material with a radiodensity above or below the threshold. To save the ROI and the measurements the raters used the “Save” function in the menu to the left and then clicked “Export ROIs”. “All ROIs in ‘source’” was selected. The raters wrote the desired name for the ROIs in the “Series description” and clicked next. They then chose “Send to datastore” and clicked “Send” to complete the export.

Each rater’s measurements were conducted independently without presence of any other rater. The raters were blinded to the results of the other raters until all measurements had been made. The raters were also blinded to which scans belonged in the “Low quality” and “High quality” group. Each rater was aware that their measurements would be compared with those of other raters. The raters also kept track on how much time was used on measuring Table 1. The raters were given as much time as needed to complete the 50 scans and they were not aware of how much time the other raters had used. AW analysed the same images twice with an interval of two weeks to assess intrarater agreement, when analysing the second time AW was blinded for all previous measurements. CM also analysed the same images twice but with an interval of 30 weeks. This was done to asses intrarater agreement for a less experienced rater with a longer duration between the analyses. When CM performed the analysis the second time, he was blinded to any of the prior measurements.

### Raters

When choosing the raters, we focused on choosing raters with varying levels of experience in the measuring technique. Four raters were chosen in total. Two raters had substantial experience in the measuring technique (AW and FD), having approximately 48–64 hours of prior measuring experience each. One rater (AB) had less experience, approximately 16 hours of prior experience.

The last rater (CM) was new to the technique. CM was taught the technique in approximately one hour. CM was then supervised during four trial measurements that were performed on scans which were not included in this article. CM was given the opportunity to ask AW questions during the four trial measurements. When CM analysed the scans the second time, he had not performed any µCT analysis for 30 weeks. He analysed the scans again without supervision and without any instructions. All raters focused on starting the ROI at the exact point of intersection between tibia and fibula and including all hindlimb tissue below the tibio-fibular joint in the ROI. The raters were told not to manually include tissue to the ROI. The raters were allowed to manually remove tissue from the ROI, when the ROI included irrelevant tissue such as tail or trunk due to poor positioning.

### Statistics

Descriptive statistics were applied according to data type: continuous variables were displayed by means and standard deviations (mean ± SD) or, alternatively, by medians (minimum-maximum) in case of asymmetric distributions as visually assessed by histograms with approximating normal distributions; categorical variables were summarized by frequencies and respective percentages. Box-and-whisker plots were employed for visualization purposes of raw data, and Bland-Altman Limits of Agreement were derived for both intra- and interrater variation analysis^21,22,26,27^. Bland-Altman plots were supplemented by exact 95% confidence intervals for the Limits of Agreement^28,29^ as well as lines from linear regression of the differences on the means^30^. The analyses were stratified by lymphedema and control, and subgroup analyses by the quality of the scanning as judged by rater 1 (AW), i.e. low vs. high, were done to illuminate consequences of scanning quality on intra- and interrater agreement. The sample size of 50 mice was motivated by a former broad recommendation of 50 subjects with three replicates on each method in a method comparison study^31^. Here, 50 scans are sufficient to conclude with 80% power and a significance level of 5% (one-sided) that the population standard deviation of paired differences is smaller than 1.92, assuming a standard deviation of 1.5^32^. All analyses were performed by using STATA/MP 15.1 (StataCorp, College Station, TX 77845, USA).

## Supplementary information

Supplementary figure S1-S4.

Dataset 1.

## Data Availability

All data generated or analysed during this study are included in Supplementary Information files.
