# Supplementary material for: Quantification of tissue volume in the hindlimb of mice using microcomputed tomography images and analysing software
Source: Sci Rep. 2020 May 19;10:8297. doi: 10.1038/s41598-020-65214-7 (PMC7237686; doi:10.1038/s41598-020-65214-7)
Supplement: Supplementary file 1 — Supplementary figure S1-S4. [file 41598_2020_65214_MOESM1_ESM.pdf]

Quantification of tissue volume in the hindlimb of mice using microcomputed tomography images and analysing software.

Alexander Wiinholt BSc[1], Oke Gerke cand.scient.oecon., PhD[2,4], Farima Dalaei MD[1], Amar Bučan MD[1], Christoffer Bing Madsen BSc[1], Jens Ahm Sørensen MD, PhD[1,3]

[1]Research Unit for Plastic Surgery, Odense University Hospital, Odense, Denmark; University of Southern Denmark, Odense, Denmark.
[2]Research Unit for Clinical Physiology and Nuclear Medicine, Department of Clinical Research, University of Southern Denmark, Campusvej 55, 5230 Odense, Denmark
[3]Department of Plastic Surgery, Odense University Hospital, J. B. Winsløwsvej 4, Odense, Denmark
[4]Department of Nuclear Medicine, Odense University Hospital, Kløvervænget 47, Odense, Denmark

This document contains:

Supplementary figure S1

Supplementary figure S2

Supplementary figure S3

Supplementary figure S4

Supplementary figure S1: Bland-Altman plots for intrarater agreement in images of low quality (n=20)

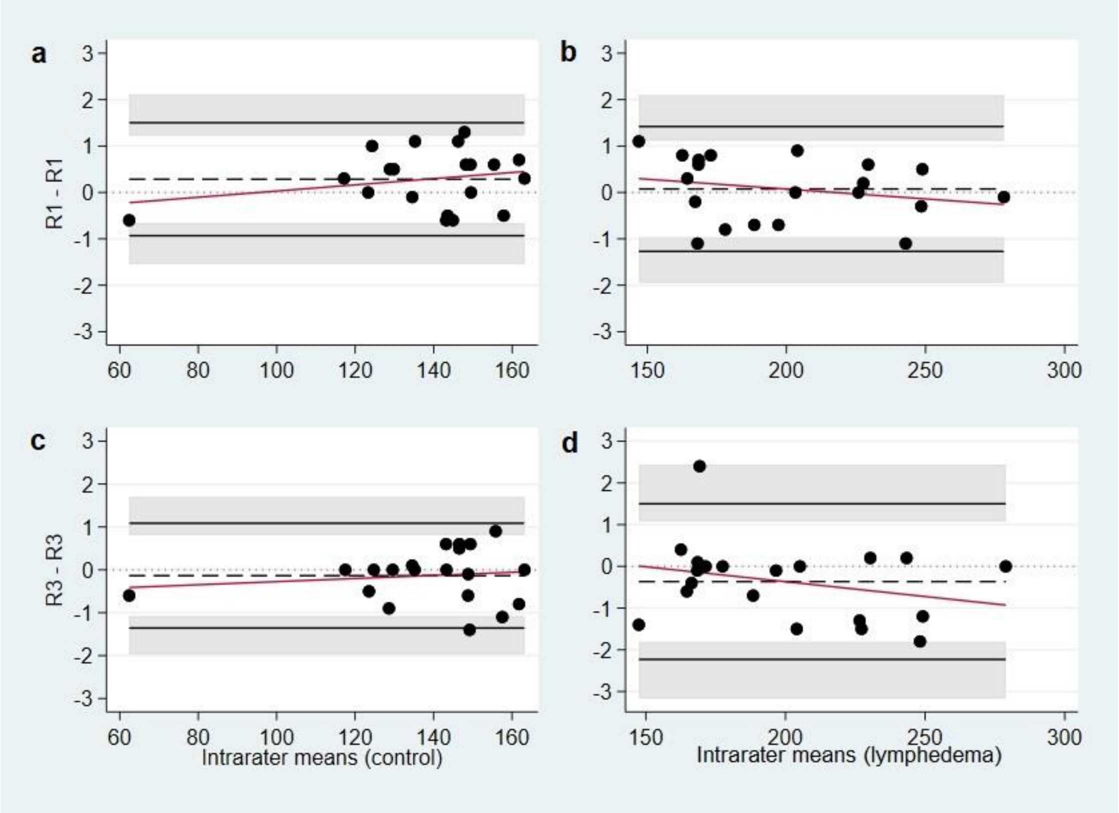

Supplementary figure S1: Bland-Altman plots for intrarater agreement in images of low quality (n=20)

The x-axis represents mean hindlimb volume in mm^3, the y-axis represents intrarater differences in mm^3.
Figure 2a and b represent data from rater 1 with “a” being data from control hindlimbs and “b” from lymphedema hindlimbs. The two analyses were performed with an interval of 2 weeks.
Figure 2c and d represent data from rater 3 with “c” being data from control hindlimbs and “d” from hindlimbs with induced lymphedema. The two analyses were performed with an interval of 30 weeks.
The grey areas represent 95% confidence intervals for the Limits of Agreement.
The red line is a linear regression line of the differences on the means.

Supplementary fig. S2: Bland-Altman plots for interrater agreement in images of low quality (n=20)

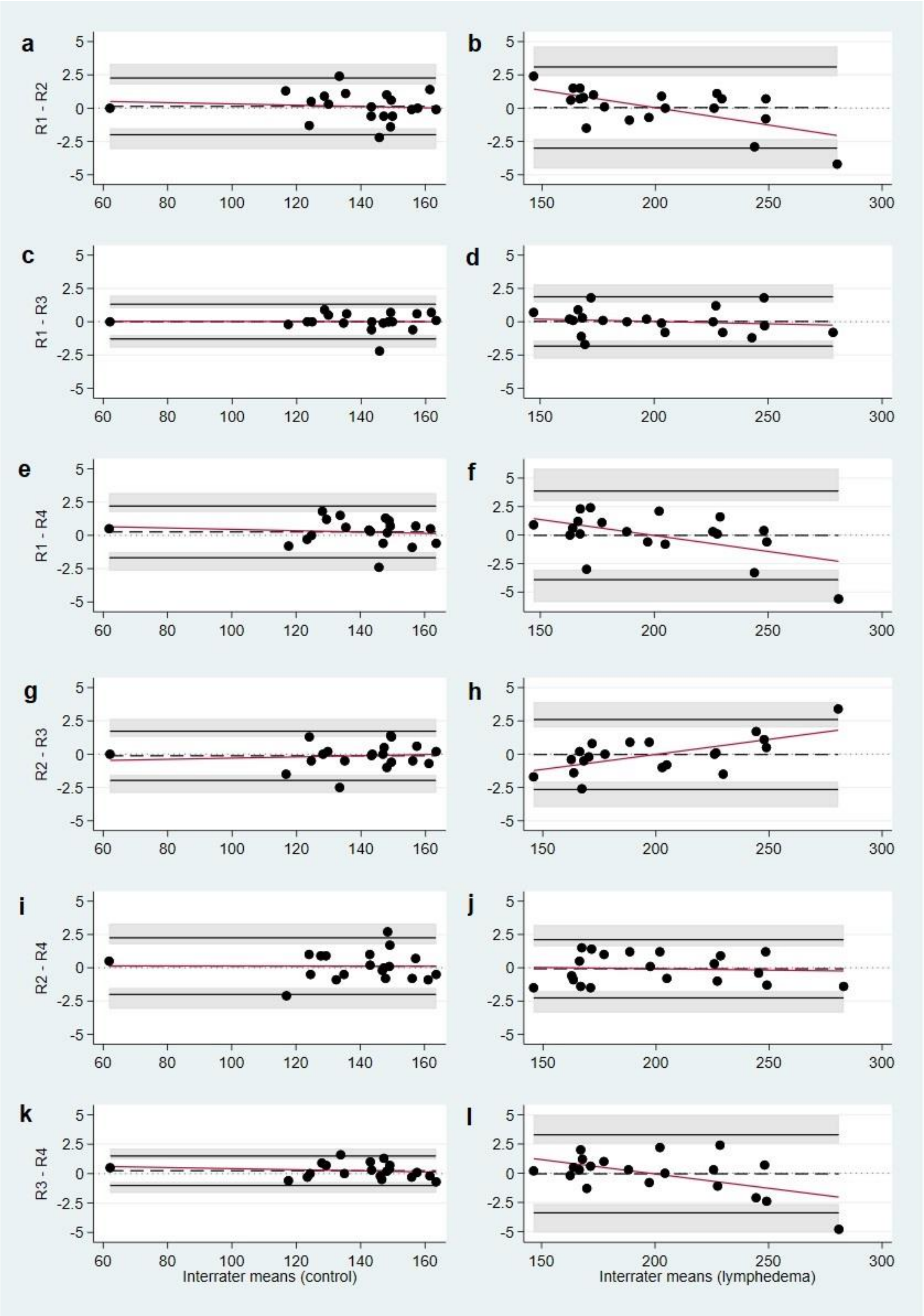

Supplementary fig. S2: Bland-Altman plots for interrater agreement in images of low quality (n=20)

The x-axis represents mean hindlimb volume in mm^3, the y-axis represents the interrater difference in mm^3. R1-R4 represent the four raters.
Figure 4a,c,e,g,i,k show data from control hindlimbs.
Figure 4b,d,f,h,j,l show data from hindlimbs with induced lymphedema.
The grey areas represent 95% confidence intervals for the Limits of Agreement.
The red line is a linear regression line of the differences on the means.

Supplementary figure S3

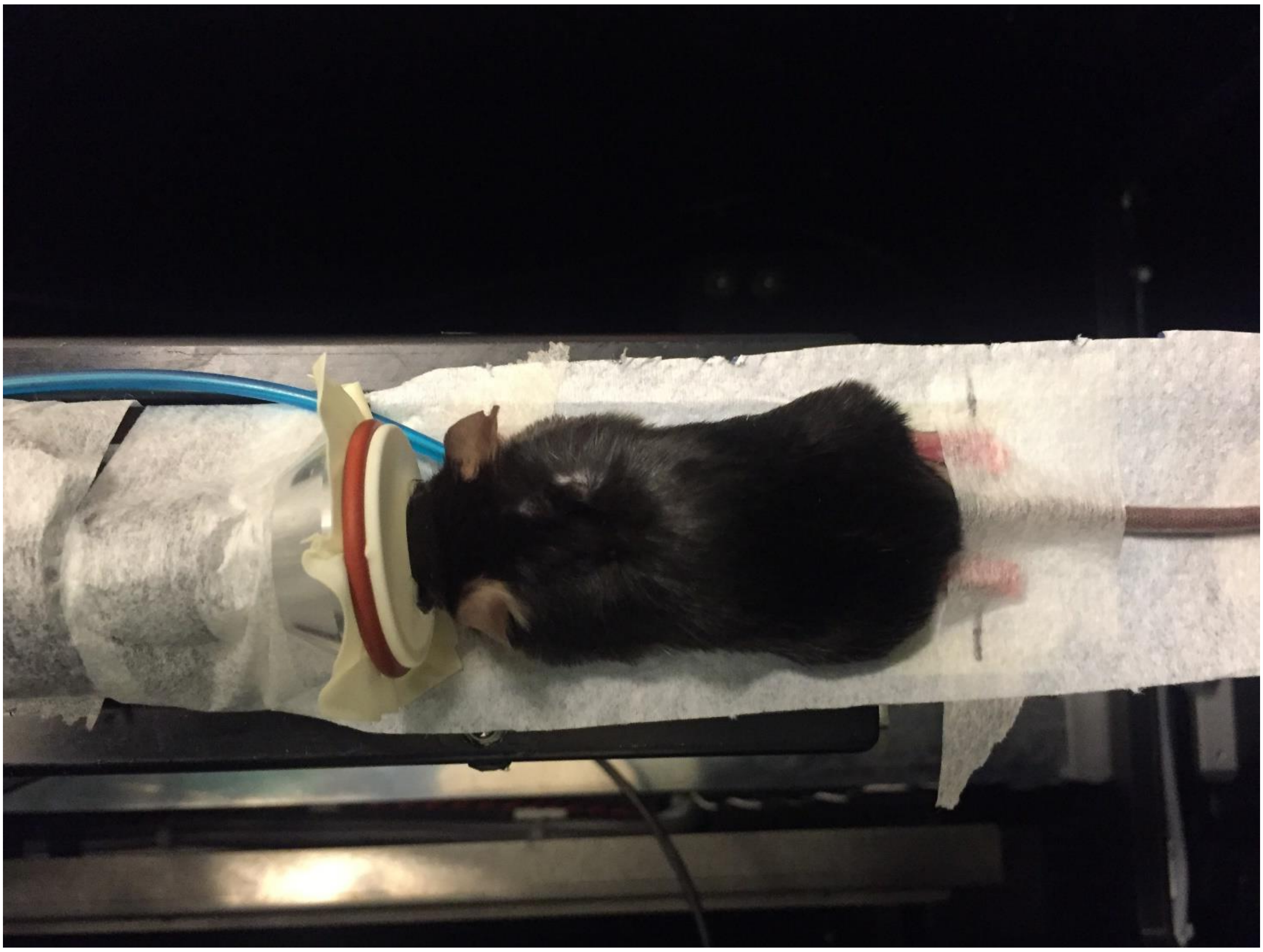

Supplementary figure S3: Example of a mouse positioned in the µCT-scanner
The mouse is shown lying in prone position. The hindlimbs and tail are stretched and fixated with surgical tape.

Supplementary figure S4

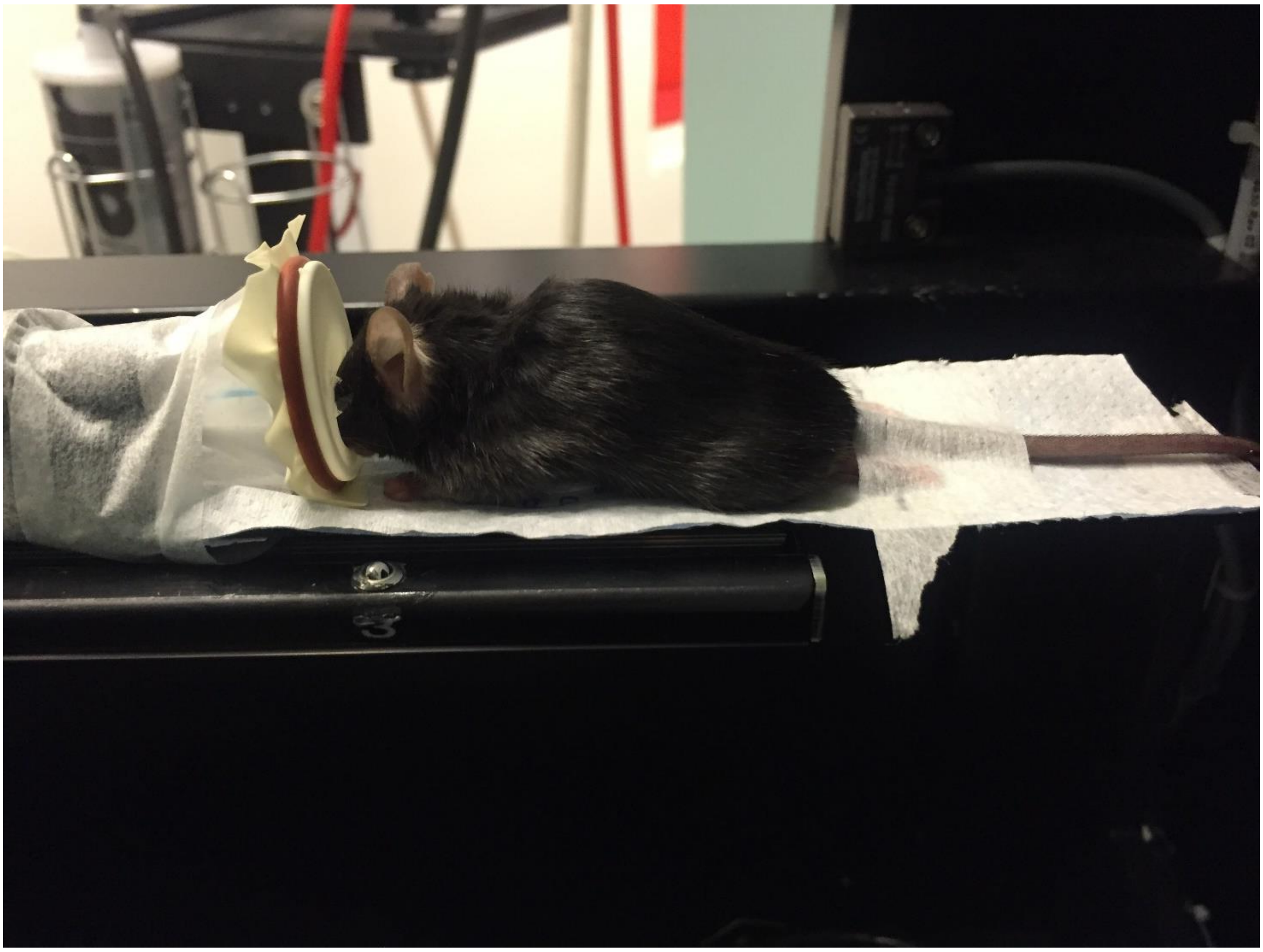

Supplementary figure S4: Example of a mouse positioned in the µCT-scanner
The mouse is shown lying in prone position. The hindlimbs and tail are stretched and fixated with surgical tape.
